# The extracellular matrix protein type I collagen and fibronectin are regulated by β-arrestin-1/endothelin axis in human ovarian fibroblasts

**DOI:** 10.1186/s13046-025-03327-5

**Published:** 2025-02-21

**Authors:** Ilenia Masi, Flavia Ottavi, Valentina Caprara, Danila Del Rio, Martina Kunkl, Francesca Spadaro, Valerio Licursi, Loretta Tuosto, Anna Bagnato, Laura Rosano’

**Affiliations:** 1 Institute of Molecular Biology and Pathology (IBPM), National Research Council (CNR), Rome, Italy; 2 Unit of Preclinical Models and New Therapeutic Agents, IRCCS, Regina Elena National Cancer Institute, Rome, Italy; 3 Department of Biology and Biotechnologies “Charles Darwin”, Sapienza University, Rome, Italy; 4 Neuroimmunology Unit, IRCCS Santa Lucia Foundation, Rome, Italy; 5 Confocal Microscopy Unit, Core Facilities, Istituto Superiore di Sanità, Rome, Italy

**Keywords:** Fibroblasts, Type I collagen, Fibronectin, Endothelin-1, Ovarian cancer, Endothelin receptors, β-arrestin 1

## Abstract

**Background:**

The invasive and metastatic spread of serous ovarian cancer (SOC) results from the cooperative interactions between cancer and stroma, which include extracellular matrix (ECM) and cellular components, including cancer-associated fibroblasts (CAFs). Soluble factors secreted by cancer and stromal cells contribute to stroma remodeling through the secretion of ECM proteins, providing a favorable environment for cancer cell dissemination. The peptide endothelin-1 (ET-1), through two G protein-coupled receptors (GPCR), endothelin receptor type A (ET_A_R) and B (ET_B_R), acts on both cancer and stromal cells, engaging the protein β-arrestin1 (β-arr1), to bolster SOC progression. However, its role in the regulation of the ECM proteins by ovarian fibroblasts is not understood. This study delves into the role of ET-1 as a regulator of type I collagen (Col1) and fibronectin (FN).

**Methods:**

We used human primary ovarian fibroblasts (HOFs) and CAFs. The expression of Col1 (*COL1A1*) and FN (*FN1*) were detected by western blotting (WB), quantitative real time-polymerase chain reaction (qRT-PCR), immunofluorescence (IF), and confocal laser scanning microscopy (CLSM) in cells and tumor tissue sections from mice xenografts, while the transcription of *COL1A1* was detected by luciferase reporter gene assay. The nuclear function of β-arr1 was evaluated by silencing and rescue expression with wild-type (WT) and nuclear mutant plasmid constructs, RNA seq and differential gene expression and gene sets enrichment analyses. The prognostic role of *COL1A1*,* FN1*,* EDN1* (ET-1) and *ARRB1* (β-arr1) gene expression was evaluated using the Kaplan–Meier plotter database and clinical ovarian cancer tissue samples.

**Results:**

We demonstrated that ET-1 boosts Col1 and FN expression in HOFs, akin to ovarian CAF levels. Both receptors are implicated, evident from inhibitory effects after ET_A_R or ET_B_R antagonist treatments and notably with bosentan, a dual antagonist, in vitro and in vivo. At the molecular level, ET-1 triggers the activation of *COL1A1* promoter activity and its enhanced expression via β-arr1 nuclear function. Transcriptome analysis of β-arr1-silenced HOFs confirms the nuclear role of β-arr1 in collagen and ECM remodeling-related protein transcriptional regulation. Accordingly, a high level of *EDN1/ARRB1* expression in combination with either *COL1A1* or *FN1* is associated with the poor prognosis of SOC patients.

**Conclusions:**

These findings hint at ET-1 involvement in ECM remodeling and early SOC stages by modulating the expression of Col1 and FN. Targeting ET-1 signaling with ET_A_R/ET_B_R antagonists might interfere with the ability of CAFs to produce key ECM proteins in this tumor.

**Supplementary Information:**

The online version contains supplementary material available at 10.1186/s13046-025-03327-5.

## Introduction

Recent studies have highlighted the crucial role of the tumor microenvironment (TME) in driving the onset and advancement of human cancer [1]. The TME is a diverse milieu comprising non-cancerous host cells like fibroblasts, endothelial cells, neurons, adipocytes, adaptive and innate immune cells, and non-cellular elements, such as the extracellular matrix (ECM). The ECM, a complex network of proteins with various soluble factors like chemokines, cytokines, growth factors, and extracellular vesicles, plays a pivotal role in supporting cancer growth and progression [2].

Within the superfamily of G protein-coupled receptors (GPCRs), the endothelin receptor type A (ET_A_R) and type B (ET_B_R) play critical roles in numerous physiological and pathological conditions, including fibrosis and cancer [3]. The ligands for these receptors, ET-1, ET-2, and ET-3, have specific functions and can cooperate in guiding tumor growth and progression, particularly in the context of human cancer within the specific TME [4].

In serous ovarian cancer (SOC), ET_A_R activated by ET-1 orchestrates various tumor-associated processes such as proliferation, apoptosis, epithelial-to-mesenchymal transition, and invasion, thereby supporting metastasis and resistance to therapeutics [4]. Additionally, ET-1 in the ovarian TME influences the activities of stromal cells involved in angiogenesis, lymphangiogenesis, immune response and the interaction of cancer cells with niche-specific stromal cells like mesothelial cells, promoting stromal invasion [4–9]. Many of these activities require the functional cooperation of ET-1 receptors with the β-arrestin1 (β-arr1) protein, in both cytosolic and nuclear compartments [5–10]. In the nucleus, the function of β-arr1 has been appreciated to regulate gene expression by binding the transcription factors and transcriptional coactivators supporting cancer progression [11–14].

Cancer-associated fibroblasts (CAFs) have emerged as key players in the complex TME, engaging in direct interactions with cancer cells during tumor progression [15, 16]. Under the influence of biochemical and mechanical cues from the TME, CAFs can differentiate into subsets that either promote or inhibit tumor growth by modulating ECM composition and integrity [17]. Collagens, a major component of the ECM, play a crucial role in cancer by interacting with specific cellular receptors, such as integrins and Discoidin Domain Receptors (DDRs), or by modifying ECM mechanical properties [18, 19]. Fibronectin (FN), another significant ECM component, serves as a prognostic marker in ovarian cancer and promotes cell invasion and migration [20, 21]. It is secreted by human mesothelial cells to enhance metastasis in ovarian cancer and can facilitate metastasis in 3D cultures by promoting cancer cell attachment to secondary organs [21–23].

Previous studies have shown that ET-1 activation of both ET_A_R and ET_B_R stimulates fibroblast activities like proliferation, motility, adhesion, myofibroblast differentiation, and collagen matrix contraction [7, 24–27]. ET-1 exerts cell-type-dependent effects on fibroblast procollagen metabolism [28]. ET-1 also influences FN and collagen deposition, contributing to fibrotic effects in diseases like cardiac fibrosis, systemic sclerosis, and cancer [29–32].

This research aims to explore how ET-1 influences ECM remodeling through the molecular regulation of type I collagen (Col1) and FN expression in human ovarian fibroblasts (HOFs) and ovarian CAFs, potentially impacting ECM-induced SOC progression.

## Materials & methods

### Cells

Primary HOFs were obtained from ScienCell (Cat# 7330-SC) and cultured in Fibroblast Medium (Cat# 2301-SC) supplemented with 10 ml of fetal bovine serum (FBS, Cat. No. 0010), 5 ml of fibroblast growth supplement (FGS, Cat. No. 2352), and 5 ml of Antibiotic solution (P/S, Cat. No. 0503).

Primary human ovarian CAFs were obtained from CTI Biotech (Cat# CTICC1.33) and cultured in Vitro Plus III, Low Serum complete MSC medium (Cat# No. PC00B1; Vitro Biopharma). Cells were incubated at 37 °C in a humidified atmosphere containing 5% CO2 and were tested for the absence of viral/mycoplasma contamination.

### Antibodies and chemical reagents

Primary antibodies (Abs) used for Western blotting (WB) were as follows: anti-COL1 (Cat# sc-293182; Santa Cruz); anti-FN (Cat# ab2413; Abcam); anti-smooth muscle actin (α-SMA) (Cat# NCL-L-SMA; Leica); anti-fibroblast-activated protein (FAP) (Cat# 28244; Abcam), anti-PDGFR (Cat# 32570; Abcam); anti-Vimentin (Cat# D21H3, Cell Signaling); anti-Tubulin (cat# sc-32293; RRID: AB_628412; Santa Cruz) anti-GAPDH (Cat# G945; RRID: AB_10597731; Sigma-Aldrich); anti-FLAG (F1804 Sigma-Aldrich); anti-β-arr1 (Cat# ab32099; Abcam). Primary Abs used for immunofluorescence (IF) were as follows: anti-COL1 (Cat# sc-293182; Santa Cruz). Secondary Abs used for WB and IF were as follows: horseradish peroxidase-conjugated goat anti-rabbit (Cat# 32460, Life Technologies) or anti-mouse (Cat# PA128568, Life Technologies). Goat anti-Mouse IgG (H + L) Cross-Adsorbed Secondary Antibody, Alexa Fluor 488 (Cat#A11001; Thermo Fisher); The chemical reagents used were as follows: Alexa Fluor 594 phalloidin (Cat# A12381; Thermo Fisher); 4’,6’-diamidino-2-phenykindole (DAPI) (Cat# 1331762; Bio-Rad Laboratories); Vectashield (Cat# H-1000; Vector Laboratories); ET-1 (100 nmol/L) (Cat# E7764-1MG; Sigma-Aldrich), BQ788 (Cat# HY-15894 A, Med Chem Express), Ambrisentan (AMB) (Cat# SML2104; Sigma-Aldrich) also called (+) - (2 S) − 2-[(4,6dimethylpyrimidin-2-yl) oxy]-3-methoxy-3,3-diphenylpropanoic acid, Bosentan (BOS) (Cat# HY-A0013A, Med Chem Express). AMB, BQ788, and BOS (1 µM) were added 20 min before the addition of ET-1.

### RNA isolation and qPCR

Total RNA was extracted from cells using GENEzol™ TriRNA Pure Kit (Cat# GZXD050, Geneaid), a phenol and guanidine isothiocyanate plus spin column system for convenient purification of high-quality total RNA, according to the manufacturer’s instructions and 1 µg was used for retrotranscription (RT) using PrimeScrip RT Reagent Kit (Cat# RR037A, Takara). Quantitative real-time-PCR was performed by using the light Cycler QuantStudio 3 qPCR System (Applied Biosystem) using SensiFAST™ SYBR^®^ Hi-ROX One-Step Kit (Meridian Bioscience). Final data were obtained by using 2^−ΔΔCt^ method (Supplementary Table 1). The number of each gene-amplified product was normalized to the number of GAPDH-amplified products. The primers used are reported in Table 1.

**Table 1:** List of primers used for quantitative real-time PCR

PRIMERS	Sequence
*EDNRA* F	5’-ATCACCGTCCTCAACCTCT-3’
*EDNRA* R	5’-CAGTGGAGAGACAATTTCAATGGC-3’
*EDRNB* F	5’-ATCACCGTCCTCAACCTCT-3’
*EDRNB* R	5’-CAGATGGAGAGACAATTTCAATGGC-3’
*EDN1* F	5’-GTGTCTACTTCTGCCACCTG-3’
*EDN1* R	5’-AAGTAAATTCTCAAGGCTCTCT-3’
*COL1A1* F	5’-CCTGTCTGCTTCCTGTAAACT-3’
*COL1A1* R	5’-TCCAGGAGCACCAACATTAC-3’
*FN1* F	5’-CTGAGACCATCACCATTAG-3’
*FN1* R	5’-GGGCTCGCTCTTCTGATTATT-3’
*GAPDH* F	5′-ACATCGCTCAGACACCATG-3’
*GAPDH* R	5′-TGTAGTTGAGGTCAATGAAGGGG-3′

### Silencing and rescue expression of β-arr1

The silencing of β-arr1 (*ARRB1*) was performed using ON-TARGET plus SMART pool smart interfering RNA (siRNAs, L-011971-00), and siGENOME control pool non-targeting was used as a negative control (SCR) (Dharmacon) [7]. In brief, 3 × 10^5^ HOFs were seeded and cultured in six-well plates until they reached 30–50% confluence and transiently transfected for 48 h, using lipofectamine RNAiMAX (Cat# 13778; Thermo Fisher Scientific) reagent according to the manufacturer’s silencing.

For rescue experiments, we used pcDNA3–*ARRB1*–FLAG (wild-type), a ‘wobble’ mutant construct encoding rat *ARRB1* sequences resistant to siRNA targeting kindly provided by Dr Robert Lefkowitz (Howard Hughes Medical Institute, Duke University, Durham, NC, USA), or the nuclear mutant pcDNA3–*ARRB1–Q394L*–FLAG, plasmid construct, transfected after 24 h of silencing using LipofectAMINE 2000 reagent (Cat# 11668027; Thermo Fisher Scientific) following the manufacturer’s instructions. The total cell lysate was collected at the endpoint of each experiment and analyzed by WB to confirm efficient knockdown and rescue.

### RNA-sequencing

Total RNA was extracted and quantified as previously described. Poly-A RNA-Seq library preparation and sequencing were performed by the BGI Genomics Co., Ltd. Three replicates for each experimental group were used. The final libraries for paired-end sequencing of 100 base pairs were carried out on a BGISEQ machine with an average yield of 45.8 millions of paired reads per sample. Reads quality was evaluated using FastQC (version 0.11.8, Babraham Institute Cambridge, UK) tool, and then reads were mapped to the human NCBI GRCh38.p12 genome using HISAT aligner. The average alignment ratio of the sample comparison genome was 90.1% and a total of 17,373 genes were detected.

### Differential gene expression and gene set enrichment analyses

To identify differentially expressed genes (DEGs) gene-level normalization and differential gene expression analysis were performed using Bioconductor [33, 34] R version 4.1.2 (R Core Team, 2015) package DESeq2 version 1.34 [35]. A total of 792 genes were identified as DEGs using cut-offs of log2 Fold change of > 1 or < -1 and false discovery rate (FDR) < 0.05. Volcano plots were created using Bioconductor R package EnhancedVolcano version 1.12.0. For visualization purpose the FDR values were transformed in -log10(FDR). DEGs were assessed with a comparison of β-arr1 knockdown lines over the control line, using a moderated t-test with. DEGs were clustered by functional annotation in Gene Ontology (GO) and pathway enrichment analysis using Bioconductor R package clusterProfiler version 4.2.0 with annotation of Cellular Component of GO Database [36] and with annotation of REACTOME database [37] for pathways. The Transcription Factors enrichment analysis was performed using Enrichr method [38] with the” TRANSFAC and JASPAR PWMs” library of transcription factors genomic binding sites. The figures were obtained using the R environment with functions from ggplot2 package version 3.3.0.

### Western blotting (WB)

For WB analysis, total cells were detached by scraping, collected by centrifugation, and lysed in RIPA buffer [50 mMTris·HCL (pH 7.5), 150 mm NaCl, 1% Nonidet P-40, 0.5% sodium deoxycholate (NaDoc), 0.1% SDS] and proteases and phosphatase inhibitors (Roche). Protein concentrations were determined using the DC Protein assay (Bio-Rad Laboratories). Cell lysates were resolved on MiniPROTEAN TGX gels and transferred to nitrocellulose membranes (Bio-Rad Laboratories), followed by WB using the primary antibodies. Primary antibodies were revealed using horseradish peroxidase-conjugated goat anti-rabbit or anti-mouse Abs. Proteins were visualized by chemiluminescence (Clarity Western ECL Substrates, Bio-Rad Laboratories) by using Azure 300 (Azure Biosystems). Quantification analyses were performed using ImageJ (https://imagej.nih.gov/ij/) and reflected the relative amounts as a ratio of each protein band relative to the loading control of the lane.

### Luciferase reporter gene assay

*COL1A1*-luciferase reporter construct (*COL1A1*-luc) was obtained by subcloning the PCR-generated fragment (-801 bp to + 154 bp) from the human *COL1A1* gene promoter (NCBI Reference Sequence: NG_007400.1) [39] into KpnI-XhoI sites of the pGL3-luciferase basic vector (Cat# E1751 Promega).

The following oligonucleotides were used:

forward 5’-CGGGGTACCCAGAAGAATTGACATCCTCAA-3’;

reverse 5’- GTCTCGAGCGCAAGGCGCGATATAGAG-3’.

Cells were transiently co-transfected with 1 µg *COL1A1*-luc vector or empty control vectors (Promega) with 250 ng pSV-β-galactosidase vector (Cat# E1081 Promega). Cells in different experimental conditions were plated in a 6-well plate until they reached 30–50% confluence and transiently co-transfected with the indicated plasmids together with the pCMV-β-galactosidase vector using Lipofectamine™ 2000 reagent. Serum-free medium alone or medium containing ET-1 and/or BOS were added to the wells and incubated for 24 h at 37 °C. Reporter activity was measured by using the Luciferase assay system (Cat# E1500, Promega) quantified by using a microplate reader (CLARIOstar, BMG Labtech). Luciferase activities were normalized to β-galactosidase activity by using a microplate reader (Neo Biotech). The mean of three independent experiments performed in sextuplicate was reported.

### Immunofluorescence and confocal laser scanning microscopy (CLSM)

Cells cultured on coverslips were fixed with 4% paraformaldehyde for 10 min at room temperature, permeabilized with 0.2% Triton-X-100 and blocked with 0.1 M glycine, 1% BSA and 0.1% Tween20 in PBS for 30 min at room temperature. Samples were incubated with primary Abs in 0,5% BSA in PBS overnight at 4 °C, followed by incubation with secondary Abs conjugated with Alexa Fluor for 1 h at room temperature. Coverslips were finally mounted with a Vectashield mounting medium for fluorescence (Vector Laboratories). CLSM observations were performed with a Zeiss LSM980 apparatus, using a 63x/1.40 NA oil objective and excitation spectral laser lines at 405, 488, 543, 594 and 639 nm. Image acquisition and processing were carried out using the Zeiss Confocal Software Zen 3.1 (Blue edition). Signals from different fluorescent probes were taken in sequential scan settings and co-localization was visualized in merge images. For each image, the fluorescence intensity of the green channel was calculated using Image J by dividing the mean fluorescent intensity (MFI) by the total number of nuclei (this ratio is the reported value).

### IF and CLSM on tumor tissue sections

In vivo animal studies were performed as previously described [7]. Briefly, female NOD/SCID mice (Charles River Laboratories) were injected intraperitoneally (i.p.) with 200 µL PBS containing 3 × 10^6^ viable SKOV3-Luc + HOF-Luc cells (3:1, SKOV3:HOFs), following the guidelines for animal experimentation. One week later, mice were randomized into three different groups undergoing the following treatments for 5 weeks: (i) 200 µL Metocell (vehicle oral gavage, CTR), (ii) 200 µL Bosentan (10 mg/kg, oral gavage daily), and Ambrisentan (5 mg/kg, oral gavage daily). Upon experimental termination, mice were euthanized, and visible metastases were carefully dissected, frozen, and used for WB, and IF analysis. WB analysis was performed as described above. CLSM analysis was performed on formalin-fixed paraffin-embedded (FFPE) tissue tumor sections from NOD/SCID mice. For detection of FN and Col1 tumor sections (5 μm thick) were deparaffinized, hydrated through graded alcohols, and subjected to a Heat-induced Epitope Retrieval step by Citrate pH6 (Novus) for 3 × 3 min in microwave. Sections were washed with PBS-T (0.01% Tween 20) and blocked in PBS-BSA 3% for 60 min at 37 °C. Primary Abs, mouse anti-Col1 or rabbit anti-FN in combination with mouse anti-SMA were added in PBS-BSA-saponin (0.5% BSA-0.1% saponin) and incubated for 60 min at 37 °C. Sections were then incubated for 60 min at 37 °C with Alexa Fluor^®^ -488 F(ab)2 fragments of goat anti-mouse IgG and Alexa Fluor^®^ -594 F(ab)2 fragments of goat anti-rabbit IgG plus DAPI (Thermo Fisher Scientific) and mounted in Vectashield antifade mounting medium (Vector Laboratories). CLSM observations were performed with a Zeiss LSM980 apparatus, using a 40x/1.40 NA oil objective and excitation spectral laser lines at 405, 488 and 594 nm. Image acquisition and processing were carried out using the Zeiss Confocal Software Zen 3.3 (Blue edition). Signals from different fluorescent probes were taken in sequential scan settings; several fields for each labeling condition were analyzed and representative results were shown.

### Bioinformatics analysis

To analyze the correlation of *COL1A1* (202311_s_at), *FN*1 (202311_s_at) or the combination of *EDN1* (222802_at) and *ARRB1* (218832_x_at) mRNA expression to overall survival (OS) and progression-free survival (PFS) in ovarian cancer a cohort of serous ovarian cancer patients (stage 1 + 2 and 3 + 4) from all datasets was interrogated using the Kaplan-Meier plotter web tool (http://kmplot.com/analysis/index.php?p=service%26cancer=ovar) [40]. The ovarian cancer patients were followed up to 5 years. To determine the prognostic value, the samples were split into two groups according to the median expression of the mean signal of the genes. The mRNA expression above or below the median separates the cases into high expression and low expression. Hazard ratio (HR), 95% confidence intervals, and log-rank P were presented on the main plots. A p of < 0.05 is considered to be statistically significant.

The gene expression profiles of A*RRB1* (GSE38666) were downloaded from the Gene Expression Omnibus (GEO) database (https://www.ncbi.nlm.nih.gov/geo), a public functional genomics data repository. The annotation platform was GPL570: [HG-U133_Plus_2] Affymetrix Human Genome U133 Plus 2.0 Array platform. Gene expression analysis of 7 cancer stroma and 7 matched cancer epithelia from 18 ovarian cancer patients [41, 42].

### Statistical analysis

All the experiments were repeated at least three times, otherwise indicated. Statistical analysis was conducted using GraphPad Prism 8.0 software and the values represent mean ± SD. Graphs comparing more than two conditions were analyzed via one-way ANOVA and the variance was similar between the groups that were being statistically compared. Statistical significance was defined as *, *P* < 0.05; **, *P* < 0.01; ***, *P* < 0.001; ****, *P* < 0.0001.

## Results

### ET-1 promotes type I collagen and fibronectin expression

**Fig. 1:**
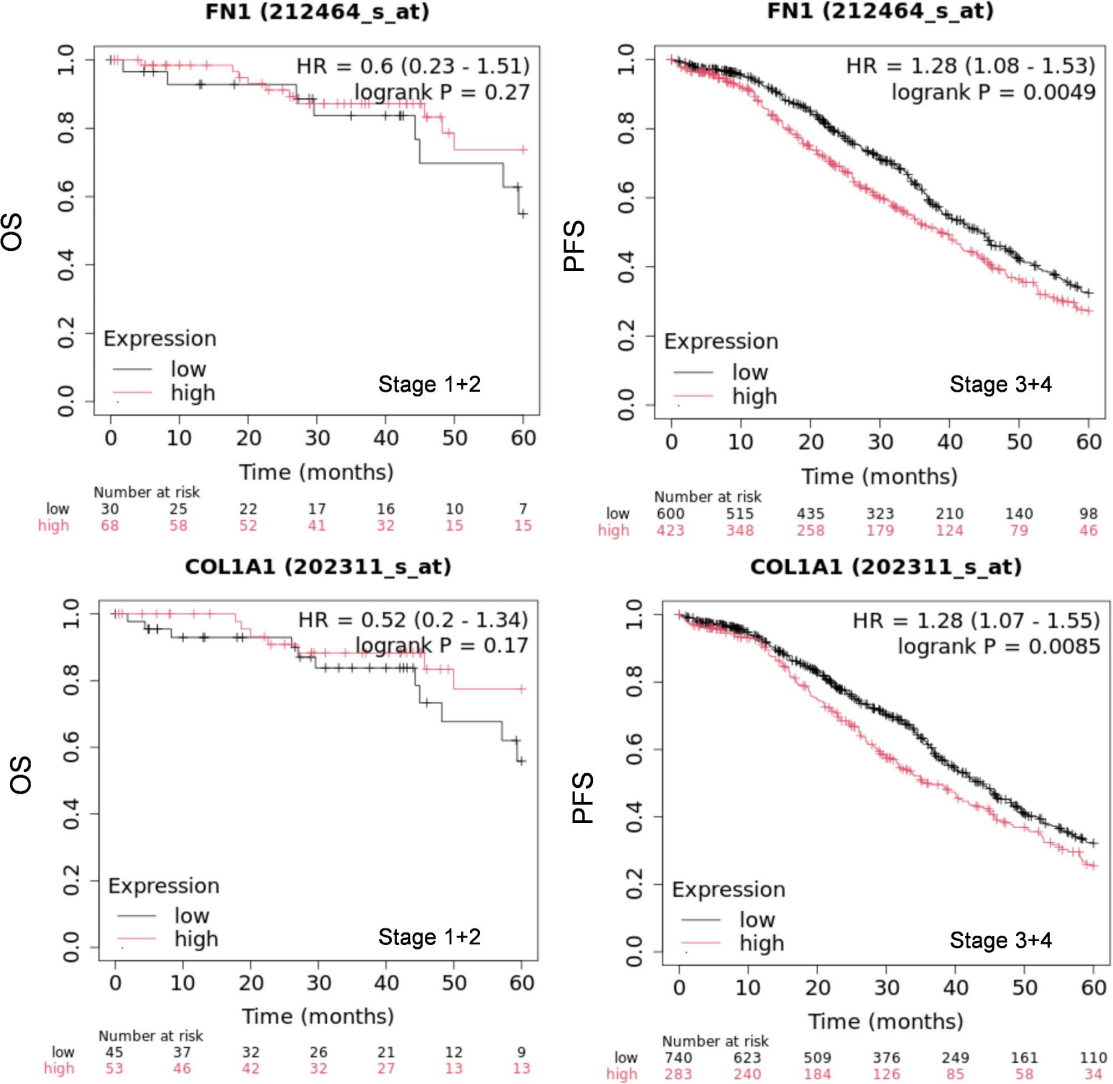
Kaplan-Meier analysis of overall survival (OS) or progression-free survival (PFS) curves in SOC patients with low or high FN1 or Col1A1 expression at stages 1 + 2 and 3 + 4

**Fig. 2:**
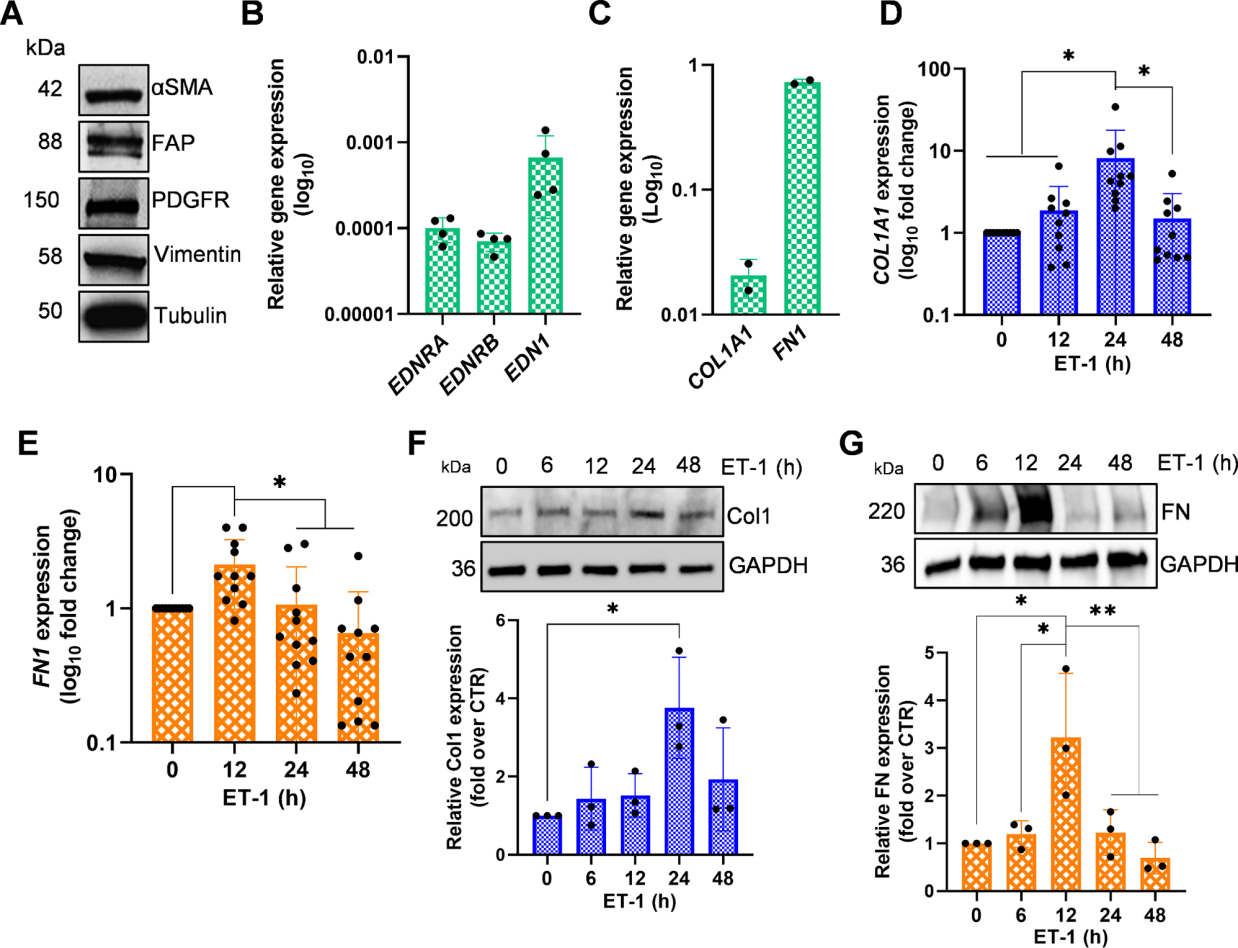
HOFs express ET-1, ET_A_R and ET_B_R, as well as *COL1A1* and *FN1*. **(A)** Western Blotting (WB) analysis of indicated protein expression. Tubulin was used as a loading control. qRT-PCR analysis of **(B)***EDNRA* (ET_A_R), *EDNRB* (ET_B_R), and *EDN1* (ET-1) or **(C)***COL1A1* (Col1) and *FN1* (Fibronectin) mRNA expression. **(D-E)** qRT-PCR analysis of *COL1A1* and *FN1* mRNA expression in HOFs stimulated with ET-1 (100 nM) at indicated time points and indicated as fold change compared to untreated cells. **(F-G)** Representative WB analysis of Col1 and FN expression in HOFs stimulated with ET-1 at the indicated time points. GAPDH was used as a loading control. Histograms, mean ± SD. *n* = 3. One-way ANOVA, *n* = 3 (*) *p* < 0.05, (**) *p* < 0.01, (***) *p* < 0.001

Recent data obtained dissecting ovarian CAF populations by ATAC and scRNA seq analysis revealed that the myCAF cluster containing *COL1A1* and *FN1* is the most critical for tumor growth and metastasis, associated with short-term survival [43–45]. We explored the correlation between their expression levels and their predictive value for SOC prognosis and related cancer staging, by using the online survival analysis software Kaplan–Meier plotter to generate survival curves and the log-rank test. As shown in Fig. 1, the survival rate of patients with high expression was significantly worse in stages 3 + 4 and not in stages 1 + 2, both in terms of overall survival (OS) and progression-free survival (PFS), confirming that their expression could be used as a tumor progression marker. To determine whether ET-1 signaling plays a role in ECM remodeling via specific protein expression and secretion, we employed primary HOFs that were characterized by the presence of α-smooth muscle actin (α-SMA), vimentin, fibroblast-activating protein (FAP), and platelet-derived growth factor receptor (PDGFR), confirming their identity as resident fibroblasts (Fig. 2A). HOFs express ET-1 (*EDN1*) along with both ET_A_ (*EDNRA*) and ET_B_ (*EDNRB*) receptors (Fig. 2B), consistent with our previous findings [7].

The structural organization of collagen is known to play a crucial role in the development, progression, and response to treatments in ovarian cancer [46–48]. Additionally, the presence of fibronectin is essential for ovarian cancer cells to invade and regulate various aspects of tumor advancement [49]. We cultured HOFs until they reached confluence and examined the impact of ET-1, a soluble mediator, on the expression of key ECM proteins in ovarian cancer, specifically collagen type I alpha 1 chain (*COL1A1*) and *FN1*, both of which are express by HOFs (Fig. 2C). Our results, depicted in Fig. 2D and E, reveal that ET-1 triggers a time-dependent increase in mRNA levels of both *COL1A1* and *FN1*, peaking at 24 h and 12 h, respectively, after the addition of ET-1. Moreover, ET-1 also enhances the levels of Col1 and FN proteins (Fig. 2F and G).

### Blocking ETA and ETB receptors suppress the expression of Col1 and FN

**Fig. 3:**
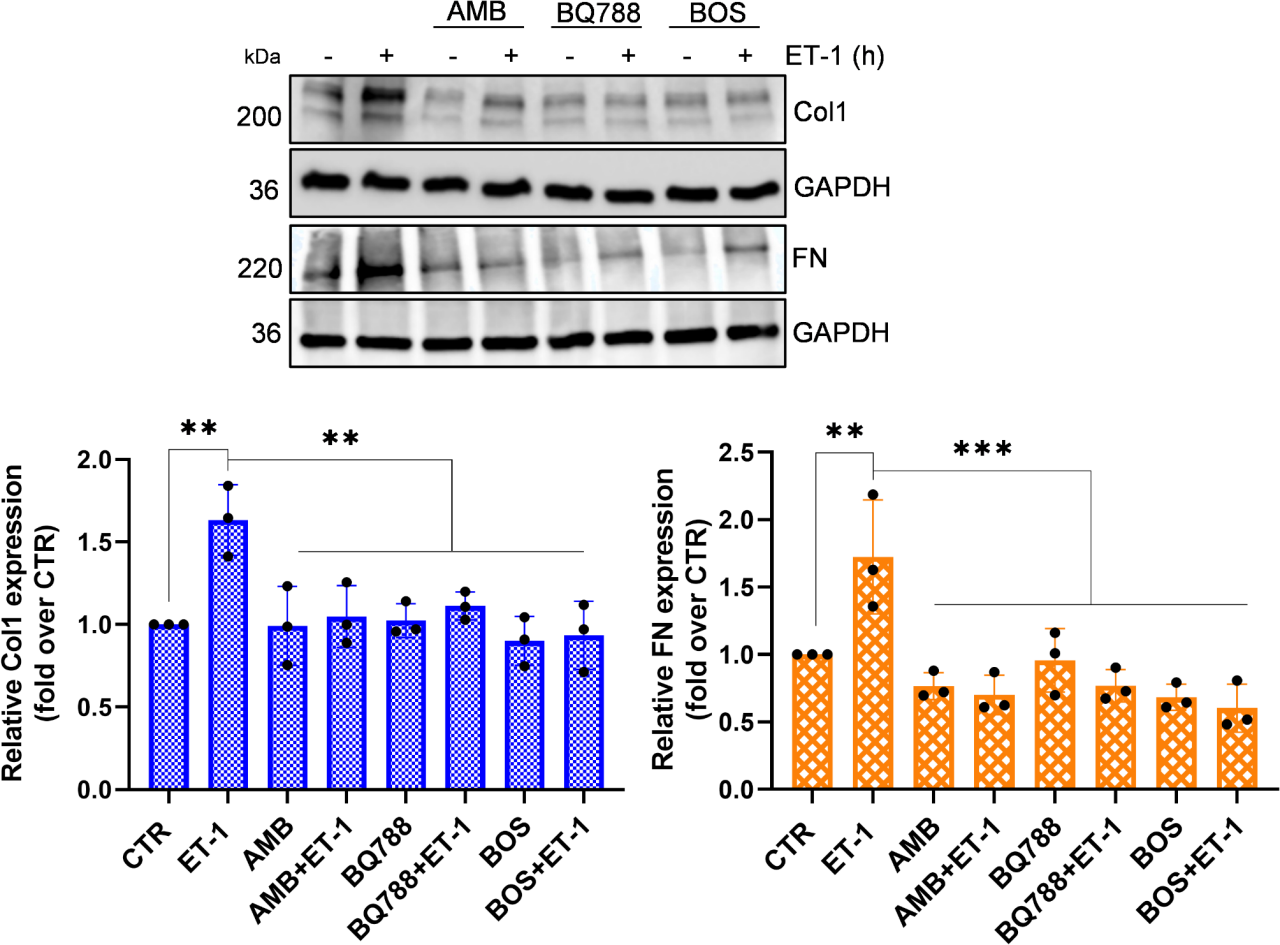
ET-1 induces Col1 and FN expression through ET_A_R and ET_B_R. Representative WB analysis of Col1 and FN protein expression in HOFs stimulated with ET-1 and/or Ambrisentan (AMB) (1 μm) and/or BQ788 (1 μm) and/or bosentan (BOS) (1 μm) for 24 h and 12 h, respectively. GAPDH was used as a loading control. Histograms, mean ± SD. One-way ANOVA (*) *p* < 0.05, (**) *p* < 0.01, (***) *p* < 0.001, (****) *p* < 0.0001

**Fig. 4:**
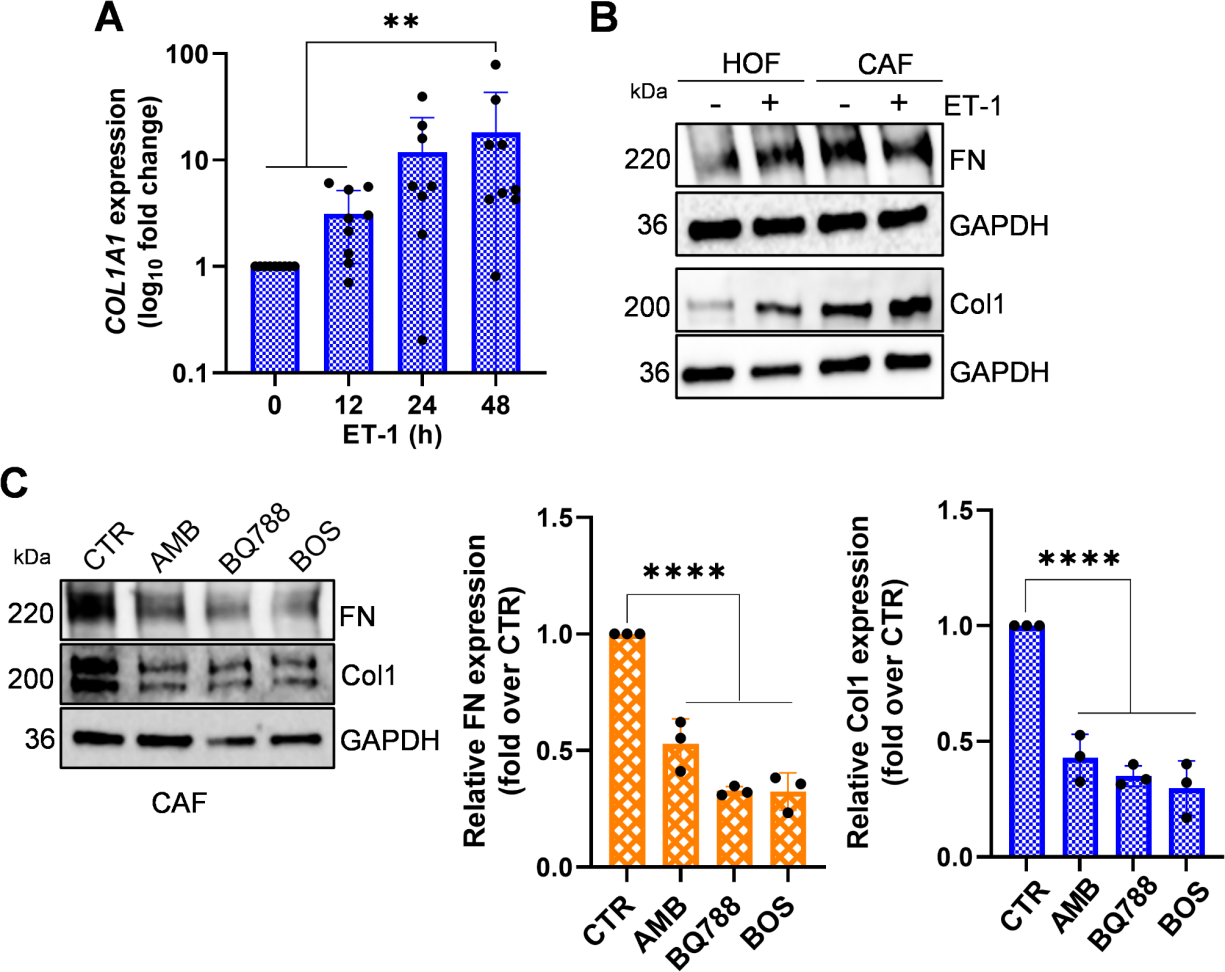
ET-1 induces the expression of *COL1A1* and *FN1* through ET_A/B_R in ovarian CAFs. **(A)** qRT-PCR analysis of *COL1A1* mRNA expression in CAFs stimulated with ET-1 (100 nM) at indicated time points and indicated as fold change compared to untreated cells. One-way ANOVA (*) *p* < 0.05, (**) *p* < 0.01, (***) *p* < 0.001, (****) *p* < 0.0001. **(B)** Representative WB analysis of Col1 and FN protein expression in HOFs and CAFs stimulated with ET-1 for 24 h. GAPDH was used as a loading control. *n* = 2 **(C)** Representative WB analysis of Col1 and FN protein expression in CAFs grown with serum-free medium (CTR) in the presence or absence of AMB or BQ788 or BOS (24 h). GAPDH was used as a loading control. Histograms, mean ± SD. One-way ANOVA, *n* = 3. (*) *p* < 0.05, (**) *p* < 0.01, (***) *p* < 0.001), (****) *p* < 0.0001

Since both ET-1 receptors are present on HOFs, we investigated the impact of receptor blockade by exposing the cells to specific antagonists: the ET_A_R antagonist, AMB, the ET_B_R antagonist, BQ788, or the dual ET_A_R/ET_B_R antagonist BOS. Pre-treatment with AMB or BQ788, and particularly BOS, resulted in a marked blockade of the ET-1-mediated up-regulation of Col1 and FN (Fig. 3), underscoring the involvement of both receptors. To assess the potential therapeutic implication of targeting ET_A_R and ET_B_R in CAFs, we conducted additional experiments. CAFs express both ET_A_R and ET_B_R [7]. The addition of ET-1 upregulated in a time-dependent manner *COL1A1* expression (Fig. 4A), but not of *FN1* (data not shown). Moreover, while CAFs did not exhibit a clear increase in Col1 and FN protein following ET-1 stimulation compared to HOFs (Fig. 4B), treatment with AMB, BQ788, particularly BOS, effectively constrained the production of these ECM proteins (Fig. 4C). This data suggests that disrupting the ET-1 autocrine pathway in CAFs could offer a promising therapeutic strategy.

### ET-1 modulates COL1A1 expression through the nuclear function of β-arr1

**Fig. 5:**
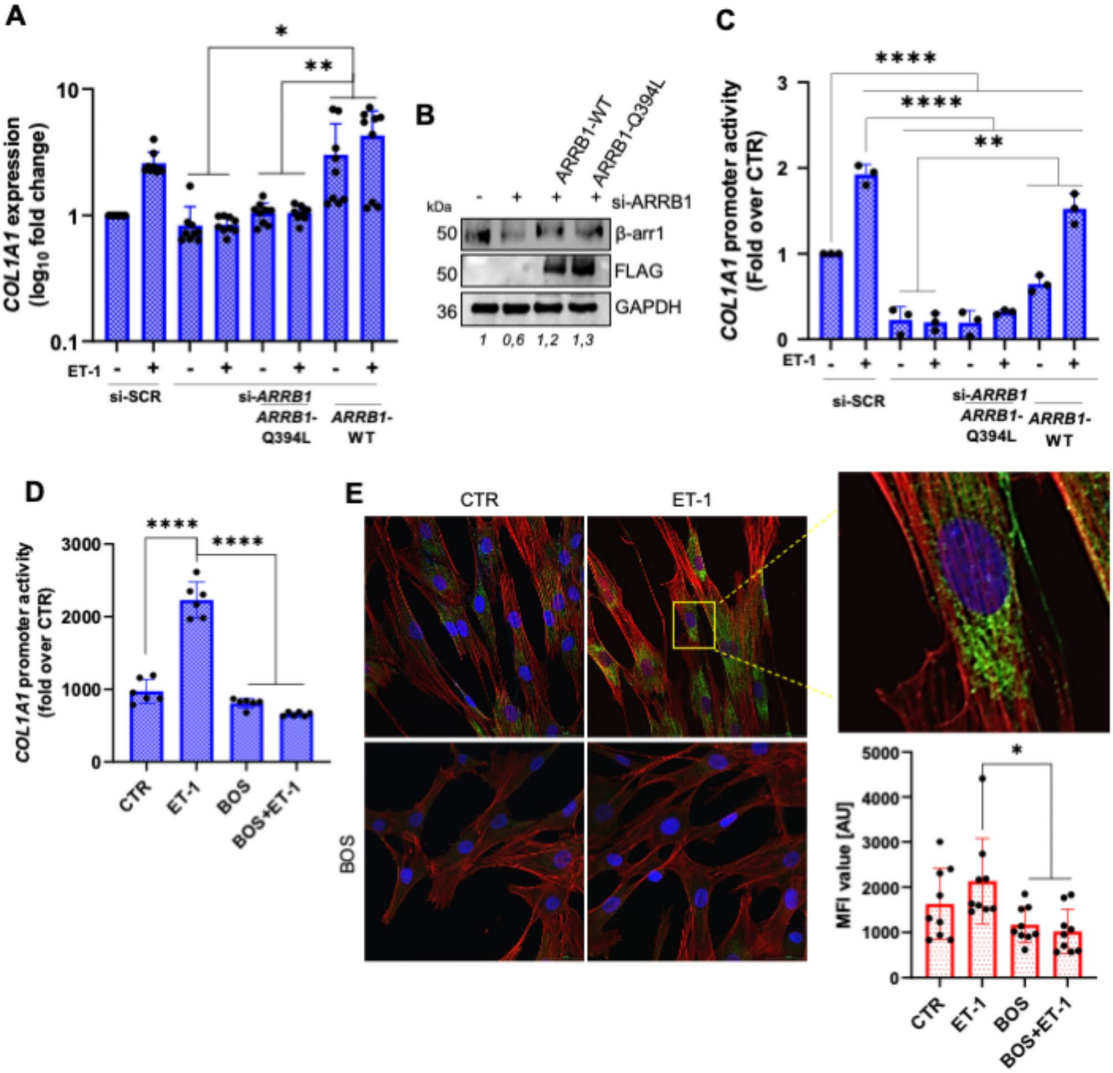
ET-1/ET_A_/ET_B_R induces expression of *COL1A1* through nuclear β-arr1. **(A)** qRT-PCR analysis of *COL1A1* mRNA expression in HOFs silenced for β-arr1 (si-*ARRB1*) and transfected with Mock or *ARRB1-Q394L-FLAG* or *ARRB1-WT-FLAG* and stimulated with ET-1 (24 h) and shown as fold over CTR. **(B)** Representative WB of whole cell lysates from HOFs transfected as in **A.** and probed with Abs to β-arr1 and FLAG. GAPDH was used as loading control. **(C)***COL1A1* promoter activity in HOFs treated as in **A.** or **(D)** in HOFs stimulated or not with ET-1 and/or BOS (24 h), calculated as Firefly Luc value/GAL enzyme activity. Histograms, mean ± SD. One-way ANOVA, *n* = 3. (*) *p* < 0.05, (**) *p* < 0.01, (***) *p* < 0.001, (****) *p* < 0.0001. **(E)** IF analysis of HOFs stimulated or not with ET-1 and/or BOS (24 h). Cells were stained for Col1 (green), F-actin (red), and DAPI (blue). Scale bar 50 μm. Histograms, mean ± SD. One-way ANOVA, *n* = 3. (*) *p* < 0.05

Given the established role of β-arr1-mediated nuclear signaling in ET-1-related functions, forming various transcriptional partnerships to enhance gene transcription [10–14], we aimed to investigate whether ET-1/β-arr1 could impact the regulation of the *COL1A1* at a molecular level. To test this hypothesis, we first examined COL1A1 expression in HOFs that had been silenced or not for β-arr1. As shown in Fig. 5A, ET-1-induced *COL1A1* expression was notably reduced in β-arr1-silenced cells, implying the involvement of β-arr1 in *COL1A1* gene regulation. In contrast to *COL1A1*, ET-1-induced *FN1* expression is not significantly reduced in β-arr1-silenced cells, thus indicating that other mechanisms control the regulation of *FN1* by ET-1 (Suppl. Figure 1). To confirm the nuclear function of β-arr1 on *COL1A1* expression, we rescued the expression of β-arr1 by introducing *ARRB1*-WT or *ARRB1*-Q394L, a mutant variant of β-arr1 carrying a single point mutation (Q394L) introducing a nuclear export signal [12]​. *ARRB1*-Q394L-transfected HOFs exhibited decreased *COL1A1* expression following ET-1 addition, which was rescued by the re-expression of *ARRB1*-WT (Fig. 5A, B). Moreover, the full-length COL1A1-luc vector, which contains the *COL1A1* gene promoter (-801 to + 154 bp), was generated and transfected into HOFs, which were then stimulated with ET-1. Luciferase reporter gene assays demonstrated a significant increase in *COL1A1* promoter activity upon ET-1 exposure, which was negated by si-*ARRB1* or pre-treatment with BOS (Fig. 5C, D). The rescued effect of re-expression of *ARRB1*-WT but not *ARRB1*-Q394L reinforces the involvement of nuclear β-arr1 in the ET-1-driven transcriptional regulation of *COL1A1* (Fig. 5C). To further validate these findings, an immunofluorescence analysis was conducted to assess Col1 expression. MFI results indicated cytoplasmic Col1 expression in HOFs (Fig. 5E). Following a 24 h ET-1 treatment, a significant increase in Col1 expression was observed, which was reversed in cells treated with BOS (Fig. 5E). These data support the notion that β-arr1 functions as a nuclear regulator of ET-1 receptor-dependent *COL1A1* expression.

### β-arr1 triggers a molecular response upregulating genes coding for ECM-related proteins

**Fig. 6:**
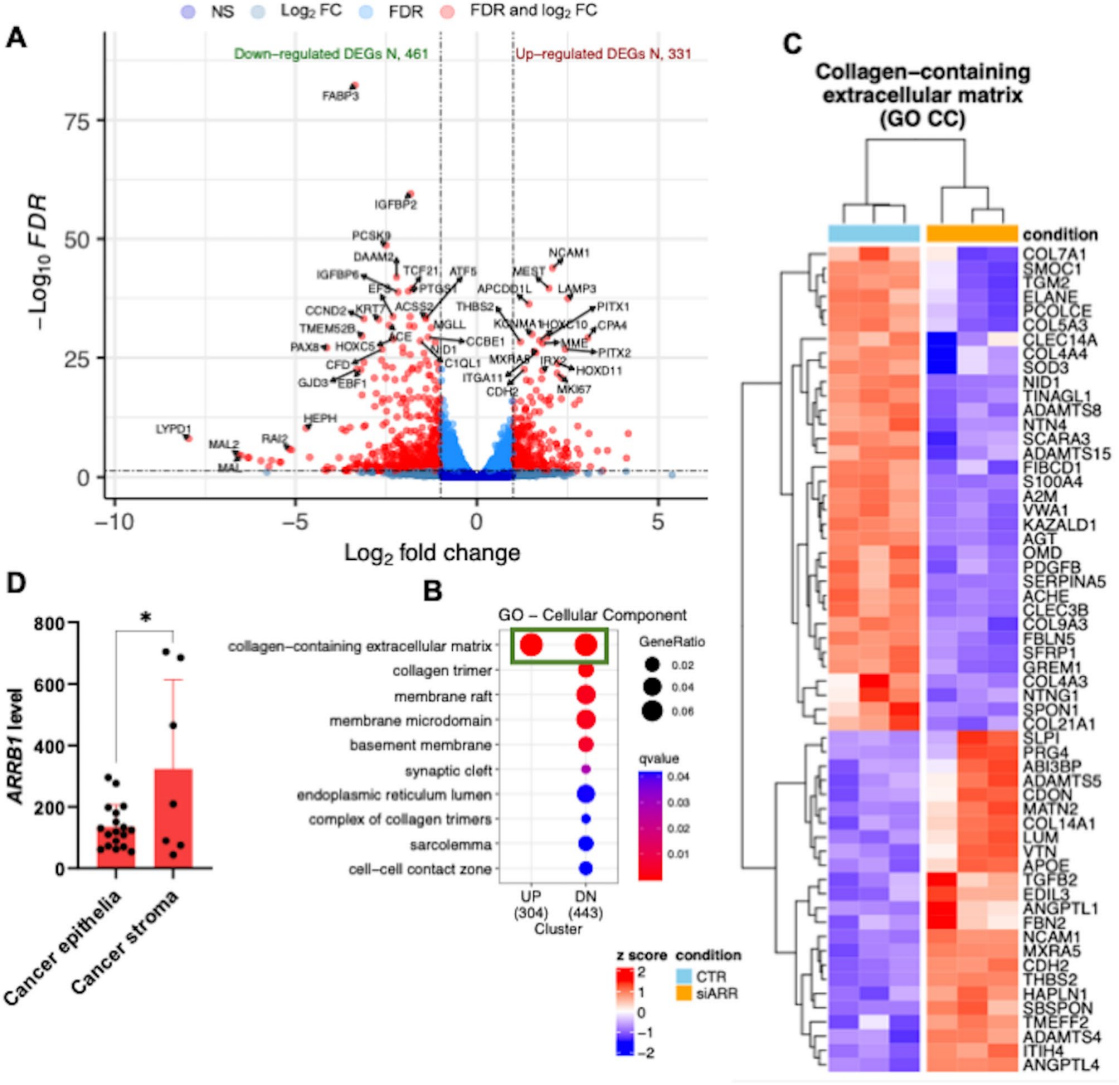
RNA-seq analysis of HOFs silenced or not for β-arr1. **(A)** Volcano plot showing genes’ −log10(FDR) and log2(fold change). Significantly modulated genes (FDR<0.05 and|log2 fold change| > 1) are highlighted in red, whereas not significantly modulated genes are indicated in dark blue. The vertical dashed lines indicate the thresholds for up- and down-regulated genes and the horizontal dashed line represents the FDR threshold. The top 50 most significant DEGs are labeled. **(B)** Dot plot of over-representation analysis of upregulated and downregulated DEGs which shows significantly enriched gene sets according to GO CC terms. Dot size indicates a ratio between the number of genes participating in the current GO CC category and the number of up or downregulated DEGs. The dot color indicates the FDR (qvalue) associated with each enriched term. **(C)** Heatmap of z-score scaled expression values of genes belongs to the collagen-containing extracellular matrix category. **(D)** Analysis of ARRB1 expression in 7 cancer stroma and 7 matched cancer epithelia from 18 ovarian cancer patients. One-way ANOVA

To validate the role of the ET-1/βarr-1 axis in matrix remodeling and desmoplasia driven by HOFs, we conducted transcriptomic profiling of HOFs transfected or not with si-ARRB1. Our RNA-seq analysis focused on identifying differentially expressed genes (DEGs) between HOF conditions (CTR and si-ARRB1). We identified 461 down-regulated and 331 up-regulated DEGs (FDR < 0.05,|Fold change| > 1, Fig. 6A), indicating a significant impact of ARRB1 down-regulation on the expression of various coding genes (Suppl. Figure 2 A). To gain insights into the molecular mechanisms mediating the effects of β-arr1 knockdown we clustered the DEGs by performing gene sets enrichment analysis using Gene Ontology [GO] and REACTOME pathway databases. Notably, our analysis revealed a significative (FDR < 0.01) cluster of DEGs enriched in genes coding for ECM-related proteins. Specifically, we identified a cluster associated with collagen-containing ECM, which includes the downregulated genes *COL7A1*, *COL5A3*, and *ADAMTS8*, alongside the up-regulated *COL14A1* and *ADAMTS5* (Fig. 6B, C, and Suppl. Figure 2B). These findings support the notion that nuclear β-arr1 initiates a molecular response that enhances ECM remodeling and collagen deposition in HOFs. In addition, we evaluated its expression from cancer stroma and matched cancer epithelium from the GSE38666 dataset downloaded from the Gene Expression Omnibus (GEO) database. As shown in Fig. 6D, the expression level of ARRB1 in cancer stroma is significantly higher than in cancer epithelia, further validating its role in SOC stroma.

### ETA/BR blockade in vivo decreases Col1 and FN expression in peritoneal metastasis

**Fig. 7:**
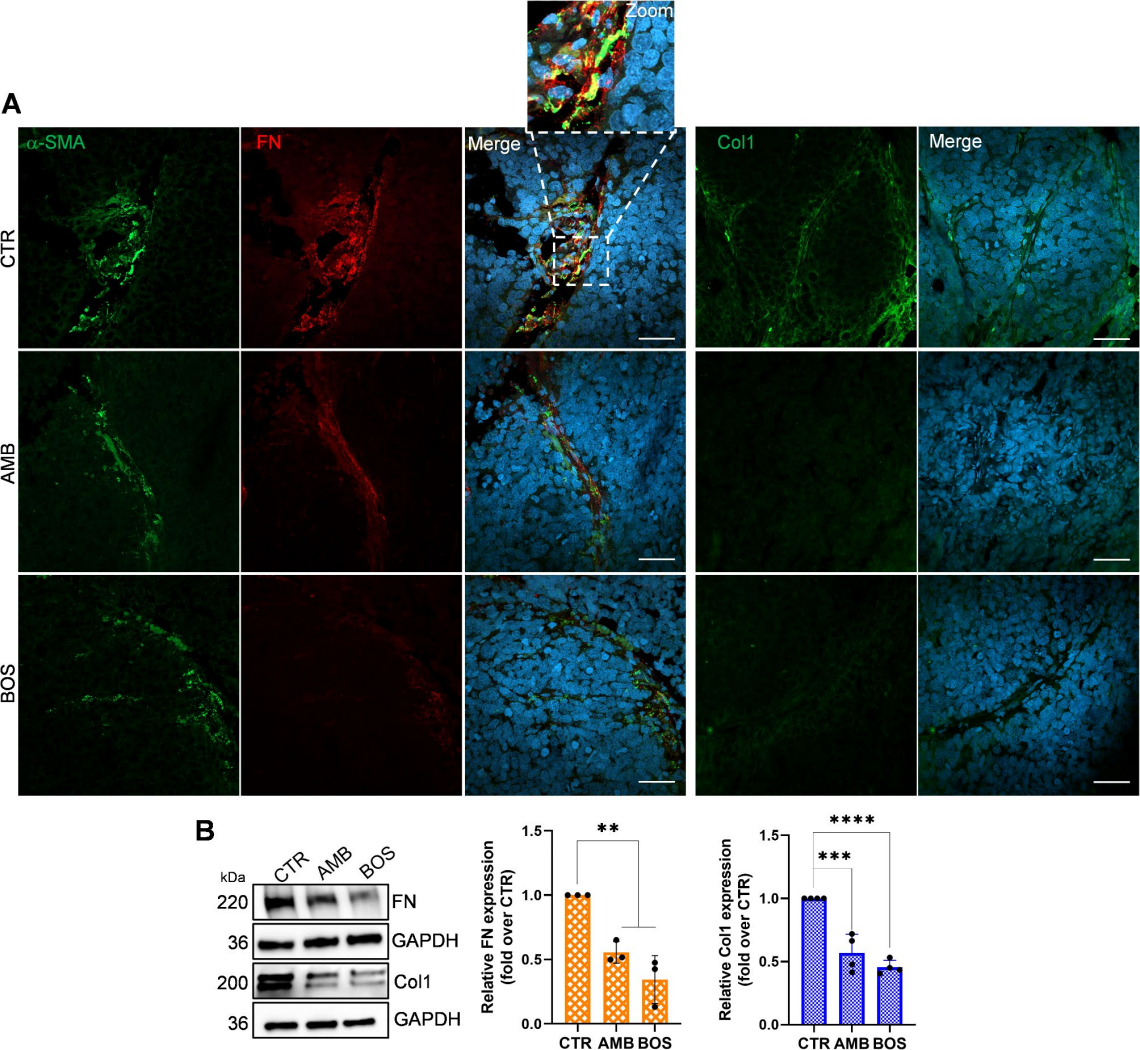
ET-1 receptor blockade inhibits the expression of Col1 and FN in SOC/HOF xenografts. **(A)** CLSM examinations (3D reconstruction images) of FFPE tissue Sect. (5 μm thick) from untreated (CTR) and AMB or BOS-treated mice. Sections were stained for FN detection (red) in combination with anti-αSMA (green, left panels) or anti-Col1 primary Ab, shown in green (right panels). Nuclei were stained with DAPI (blue). Separate channels and merged images are reported. Scale bar, 50 μm. *n* = 2. **(B)** Representative WB of whole cell lysates from metastatic nodules probed with Abs to Col1 and FN. Histograms, mean ± SD. One-way ANOVA, *n* = 3. (*) *p* < 0.05, (**) *p* < 0.01, (***) *p* < 0.001, (****) *p* < 0.0001

The results of our recent study, which examined the role of ET-1-activated CAFs in the early stages of SOC transcoelomic metastasis, indicated that the inhibition of ET-1 receptors could disrupt the interaction between SOC cells and CAFs, preventing the spread of cancer cells into the peritoneal cavity [7]. Moreover, we also found an increased apoptosis of cancer cells, endothelial cells, and fibroblasts, and reduced expression of the mesenchymal markers, such as vimentin and PDGFR [7]. In this study, we analyzed tissue sections and whole cell lysates from metastatic nodules obtained from previous treatments. IF analysis revealed that both AMB and BOS significantly downregulated the expression of FN (Fig. 7A, left panels) and Col1 (Fig. 7A, right panels), with a more efficacious effect of BOS, in comparison to CTR mice. Correspondingly, WB analysis of tumor tissues confirmed that both AMB and, more notably, BOS significantly downregulated Col1 and FN expression in vivo (Fig. 7B). These results suggest that inhibiting ET-1 receptors may limit the interaction between cancer cells and CAFs, thereby reducing ECM remodeling and the potential for metastatic growth of ovarian cancer cells.

### High EDN1/ARRB1/COL1A1 and EDN1/ARRB1/FN1 expression correlates with the poor prognosis of SOC patients

**Fig. 8:**
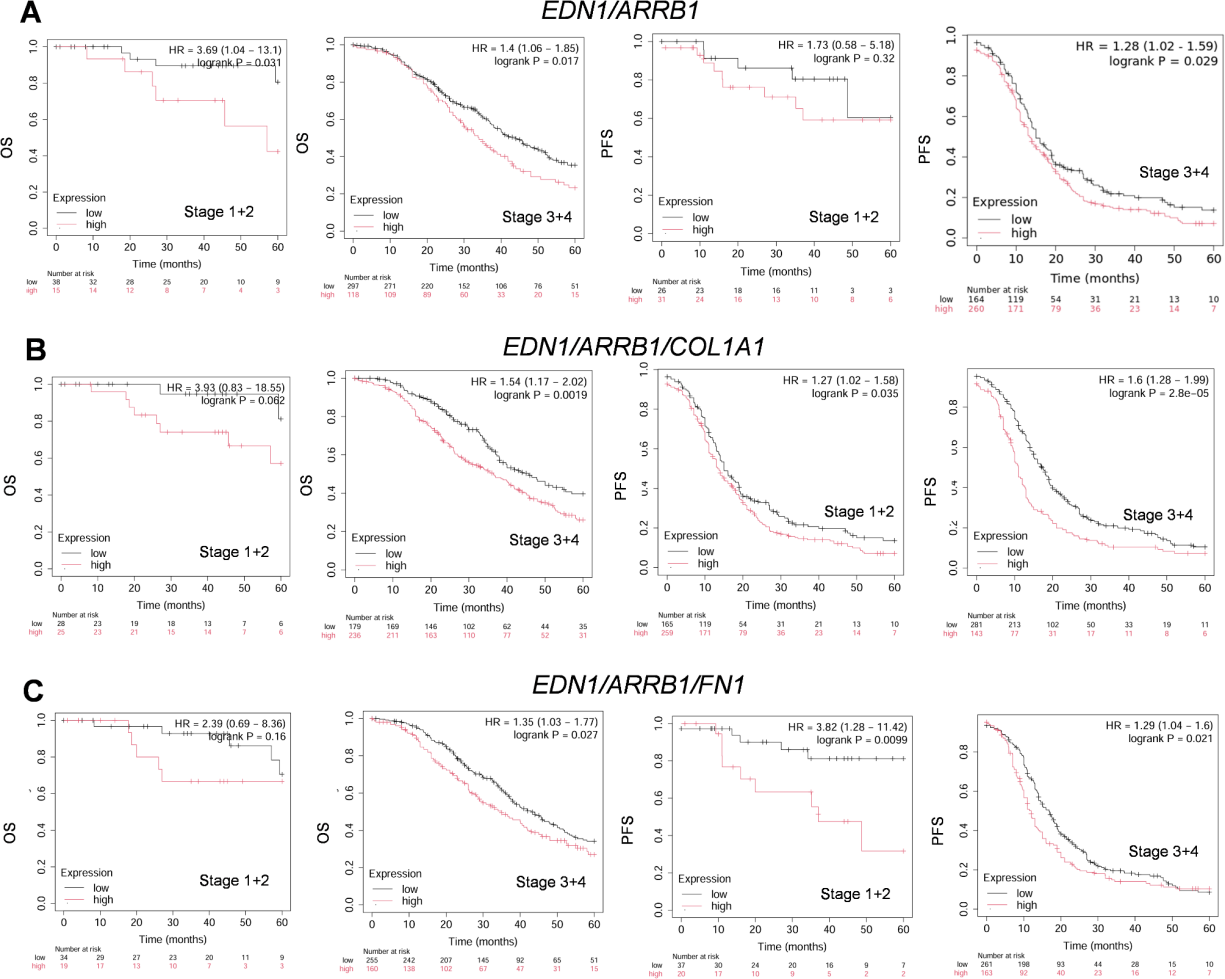
High *EDN1/ARRB1/COL1A1* and *EDN1/ARRB1/FN1* expression correlates with poor prognosis of SOC patients. Kaplan-Meier analysis of overall survival (OS) or progression-free survival (PFS) curves in SOC patients with low or high *EDN1/ARRB1***(A)** or *EDN1/ARRB1/COL1A1***(B)** or *EDN1/ARRB1/FN1***(C)** expression at stage 1 + 2 and 3 + 4

We thoroughly investigated the biological significance of *EDN1/ARRB1* expression with *COL1A1* or *FN1* by examining their expression levels and predictive value for OS or PFS and SOC cancer staging (stages 1 + 2 or 3 + 4). We generated survival curves using the Kaplan–Meier plotter and applied the log-rank test to analyze the median expression values of their combined mRNA. As shown in Fig. 8A, the survival rate of patients with high *EDN1/ARRB1* expression was significantly worse in stages 1 + 2 and 3 + 4 in terms of OS. In contrast, when considering the PFS, the survival rate was substantially worse only in the high-expression group of stage 3 + 4, suggesting a role as tumor progression markers. Further analysis of the combined expression of *EDN1/ARRB1* with *COL1A1* and *FN1* (Fig. 8B, C) revealed that although significant OS correlations were found primarily in advanced stages, a notable link between high *EDN1/ARRB1* expression and either *COL1A1* or *FN1* emerged for both early and advanced stages concerning PFS. These findings underscore the potential of these expression profiles as valuable prognostic markers in SOC.

## Discussion

OC is the seventh most common malignant tumor in women around the world [50]. Despite platinum-based chemotherapy and optimal cytoreductive surgery, this tumor is prone to metastasis and patients with OC have recurrent disease and poor prognosis. In SOC, like other solid tumors, TME and its acellular and cellular components support disease progression, revealing the emergence of novel cancer-stroma-targeted therapies [1, 47, 51]. Our findings provide evidence that the ECM protein network remodeled by CAFs may be associated with ET-1 signaling, supporting SOC progression and metastatic behavior. This regulation is linked to the activity of both receptors and might be impaired by dual receptor antagonists.

Several lines of evidence have shown that CAFs are crucial components of the TME and are involved in the growth and metastatic behavior of tumor cells by inducing proliferation, angiogenesis, and resistance to anti-tumor responses, thereby affecting various stages of cancer development [52]. HOFs have a high capacity to change their characteristics and become CAFs [53]. Considering their pivotal role within this milieu, a plethora of therapeutic modalities have been proposed for managing SOC, with a pronounced focus on the selective targeting of malignant cells and CAFs [17]. Unraveling the intricate mechanisms driving CAF generation and their activities in the TME is pivotal for deciphering their dynamic interactions with tumor cells and ECM in tumor progression. As research advances, it remains an intriguing and evolving area of study that promises to enhance our understanding of their regulation and could potentially pave the way for innovative therapeutic strategies targeting CAFs and their impact on tumor-stroma interactions.

It has been demonstrated that CAFs can promote the secretion of paracrine factors and the production of cytokines or growth factors. In addition, important functions of CAFs include the synthesis of components of the fibrillar ECM, such as collagens and FN [51]. Tumor-derived CAFs and the ECM that synthesize, together with tumor and other cell-secreted factors, constitute the tumor matrisome, which is subjected to dynamic changes that dictate cancer cell functions and response to therapy [47, 52, 53]. Specific ECM and collagen-remodeling signatures are linked to high disease scores, metastasis, and poor survival of high-grade SOC [54–60]. Since ET-1 is considered an important modulator of stromal cells [4, 61], we sought to ascertain whether ET-1 plays a pivotal role in the regulation of ovarian CAFs. Previous studies have shown that ET-1 drives fibroblastic behavior in cancer, via both ET_A_R and ET_B_R, and facilitates tumor-stroma interaction [5–9]. More recently, we demonstrated that an autocrine/paracrine ET-1/ET_A/B_R/β-arr1 axis is a key factor in the activation of ovarian fibroblast to CAFs, supporting their proliferation, the expression of CAF-specific markers, the secretion of pro-inflammatory cytokines and an increase in collagen contractility [7]. Of note ET-1 facilitates ECM remodeling by promoting the lytic activity of invasive protrusions, invadosome, and the formation of heterotypic HOF/OC spheroids with enhanced ability to invade, representing metastatic units. These functions are impaired by ET_A/B_R blockade or β-arr1 silencing [7]. Here, we provide new evidence for the role of the ET-1 axis in ovarian fibroblasts, associated with increased deposition of ECM proteins. In this context, the pro-fibrotic role of ET-1 has been documented in various diseases, involving the recruitment of critical transcription factors such as Smad, Transforming growth factor (TGF-β), and activator protein-1 (AP-1) [32, 59, 60, 62, 63]. Moreover, the pro-fibrogenic effect of the ET-1 axis was inhibited by bosentan in a pancreatic cancer model [64]. Another study revealed the involvement of the ET-1 axis in influencing microenvironmental factors during the initiation and progression of pancreatic tumors, associated with excessive accumulation of ECM proteins and inflammation [65].

The ET-1-driven ECM production might represent a way to support SOC growth and metastasis, linked to remodeled ECM in the cancer niche. This regulation is linked to the activity of both receptors since their blockade with bosentan, significantly inhibits ET-1-induced effects. The results from this study are in line with a previous study in lung cancer focusing on the interaction between fibrocytes and monocyte-derived cells, which have features of both fibroblasts and macrophages, respectively, and produce ECM proteins such as Col1, and the shift of cancer cells to a more proliferative and migratory phenotype, supporting the formation of a metastatic niche, at the same extent of fibroblasts [30]. The same authors demonstrated that the upregulation of ET-1 and its receptors in the lung cancer niche is crucial in mediating the effects of fibrocytes, while bosentan impaired the in vitro and in vivo effects, associated with decreased Col1 expression [30]. In a breast cancer model, the delivery of the dual receptor antagonist macitentan suppressed the recruitment of CAFs and the production of ECM constituents through inhibition of ET-1 signaling, which is consistent with the in vitro results [66]. Moreover, macitentan might interrupt ET-1-driven pro-survival signals, affecting not only SOC cells but also the dynamic interactions within the TME [9]. Our previous research indicated that bosentan is an effective agent for regulating the metastatic potential of HOF-based metastatic units, with a pronounced effect on apoptosis in cancer cells, fibroblasts, and endothelial cells [7].

## Conclusions

**Fig. 9:**
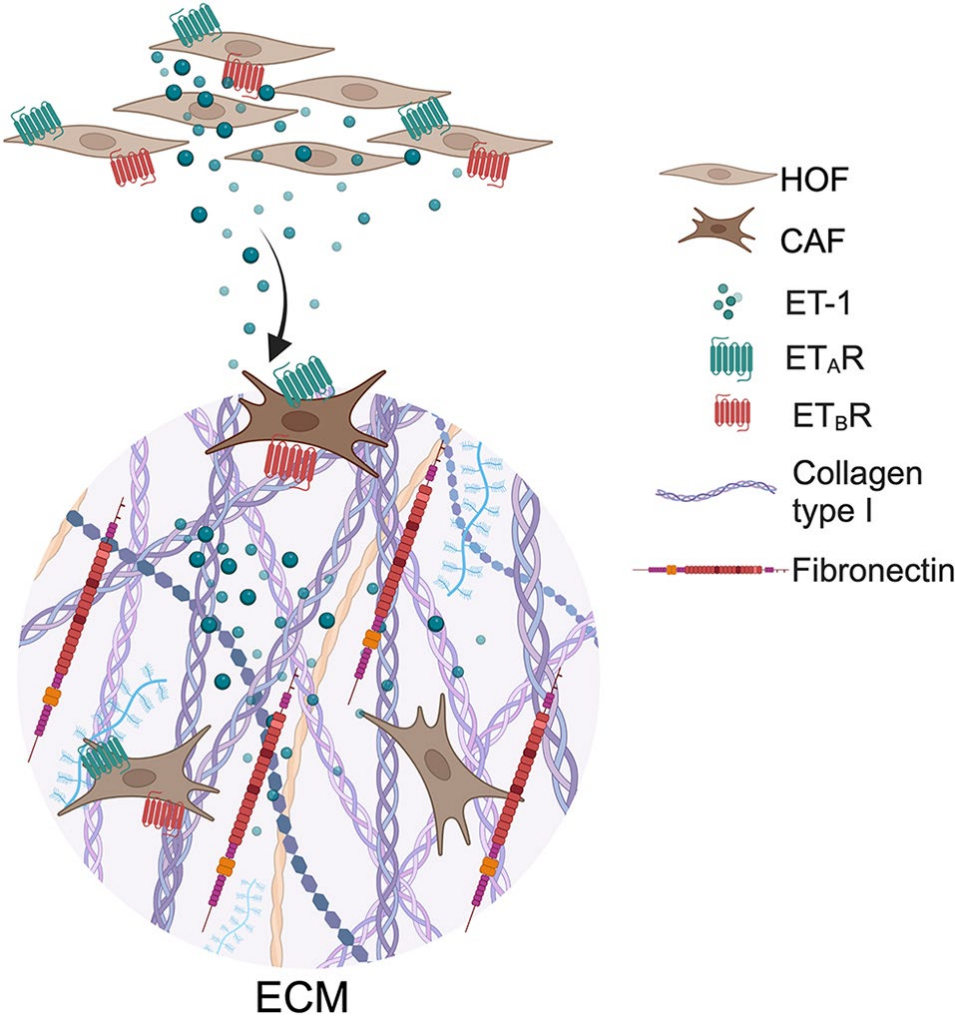
Schematic representation of proposed mechanism by which ET-1 promotes ECM protein expression and production through ET_A_R/ET_B_R axis in ovarian fibroblasts. Autocrine and paracrine ET-1 activates ET_A_R and ET_B_R in HOFs and CAFs, leading to Col1 and FN release in ovarian ECM. Created with BioRender.com (Agreement Number IK273AZ978)

In summary, our studies demonstrate for the first time that signaling from the ET-1 axis regulates the expression of both *FN1* and *COL1A1* (Fig. 9). This loop is active on both ovarian HOFs and CAFs, further supporting the idea that the ET-1 axis facilitates the reprogramming of CAFs toward ECM-remodeling/myofibroblastic phenotypes. Furthermore, we found a specific regulation of the *COL1A1* gene expression and transcriptional regulation, involving the nuclear function of β-arr1. In addition, data from transcriptome analysis indicate that β-arr1 might play a central role to ECM and matrisome component expression, contributing to reinforcing the presence of a specific cell population with mesenchymal phenotype or associated with stromal response, extensive desmoplasia, and worse survival, as also suggested by higher β-arr1 expression in cancer stroma compared to cancer epithelia. Accordingly, molecular indicators derived from bioinformatic analyses show that high expression levels of *EDN1/ARRB1* with *COL1A1* or *FN1* positively correlate with poor prognosis in the advanced stage of the tumor, conveying important prognostic information that might be translated in clinical situations in SOC patients. More recently, to identify matrix adhesion as an adaptive response, driving tumor aggressiveness via co-evolving ECM composition and sensing, it has been demonstrated that both primary tumors and solid metastases exhibit significant ECM development surrounding malignant cells, with a pronounced desmoplastic stromal reaction [47]. Notably, Col1 and FN are upregulated in both primary and metastatic tumors compared to normal tissues, contributing to the transformation of the tissue into an ECM-rich fibrotic TME [47], aligning with our analysis showing the presence of Col1 and FN in mouse peritoneal metastasis, suggesting stromal and tumor strategies for targeting pathways controlling ECM. Our findings potentiate the therapeutic concept that inhibition of ET-1 signaling with dual ET_A_R/ET_B_R antagonist blockade might selectively target tumor-supporting CAFs that impact matrisome components and ECM organization, key features of tumor initiation and progression.

## Electronic supplementary material

Below is the link to the electronic supplementary material.

Supplementary Material 1

Supplementary Material 2

Supplementary Material 3

Supplementary Material 4

## Data Availability

Data generated during the current study are included in this article and its supplementary information file and could be available on reasonable request by inquiring the corresponding author. Uncropped western blots can be seen in supplemental materials. The RNA-Seq accompanying this paper is available through NCBI’s Gene Expression Omnibus (GEO) repository, under accession number GSE272256 located at https://www.ncbi.nlm.nih.gov/geo/query/acc.cgi?acc=GSE272256.

## Abbreviations

OC: Ovarian cancer
ECM: Extracellular matrix
CAF: Cancer-associated fibroblast
ET-1: Endothelin-1
GPCR: G protein-coupled receptor
ET_A_R: The endothelin receptor type A
ET_B_R: The endothelin receptor type B
β-arr1: β-arrestin1
Col1: Type I collagen
FN1: Fibronectin
HOF: Human ovarian fibroblast
IF: Immunofluorescence
qRT-PCR: Quantitative real time-polymerase chain reaction
WB: Western blotting
CLSM: Confocal laser scanning microscopy
WT: Wild-type
TME: Tumor microenvironment
DDR: Discoidin Domain Receptor
Abs: Antibodies
DAPI: 4’,6’-diamidino-2-phenykindole
AMB: Ambrisentan
BOS: Bosentan
RT: Retrotranscription
siRNA: Small interfering RNA
DEG: Differentially expressed gene
GO: Gene Ontology
FDR: False discovery rate
OS: Overall survival
GEO: Gene Expression Omnibus
HR: Hazard ratio
α-SMA: Smooth muscle actin
FAP: Fibroblast-activated protein
PFS: Progression-free survival
TGF-β: Transforming growth factor
AP-1: Activator protein-1
