# Supplementary material for: The extracellular matrix protein type I collagen and fibronectin are regulated by β-arrestin-1/endothelin axis in human ovarian fibroblasts
Source: J Exp Clin Cancer Res. 2025 Feb 21;44:64. doi: 10.1186/s13046-025-03327-5 (PMC11844176; doi:10.1186/s13046-025-03327-5)
Supplement: Supplementary file 2 — Supplementary Material 2 [file 13046_2025_3327_MOESM2_ESM.pdf]

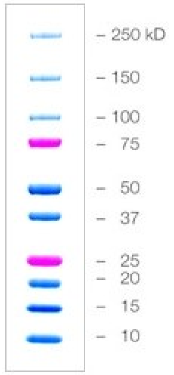

Precision Plus Protein Dual Color Standards, #1610374

Figure 2A

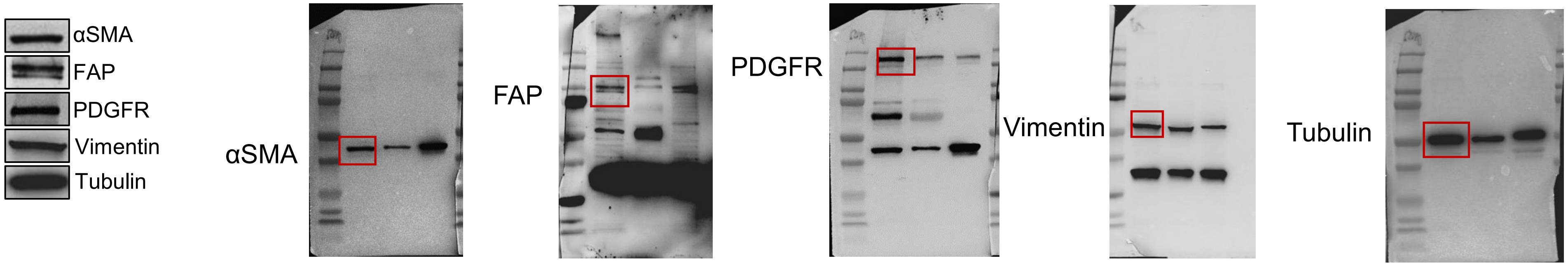

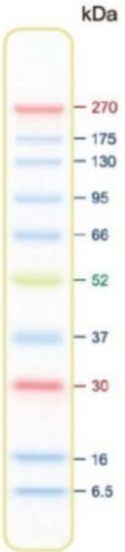

NeoPRO 10 Prestained Protein Ladder #NB-59-0002

Figure 2F

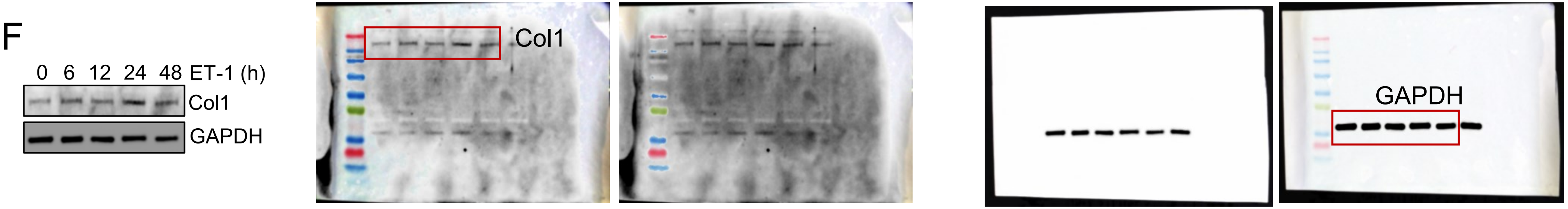

Figure 2G

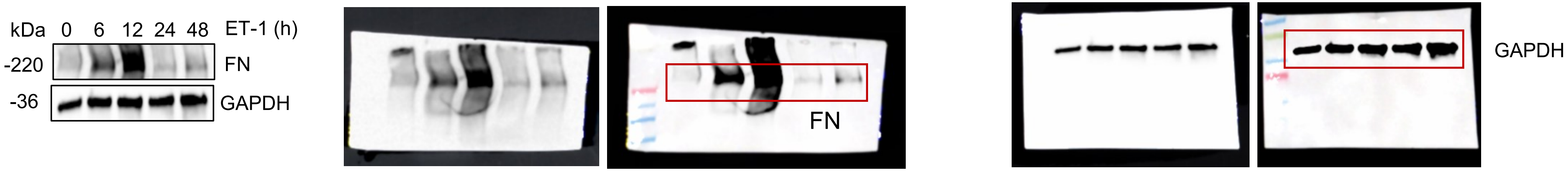

Figure 3A

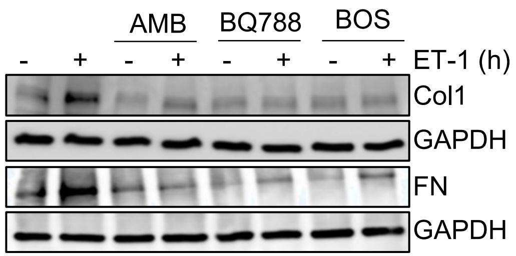

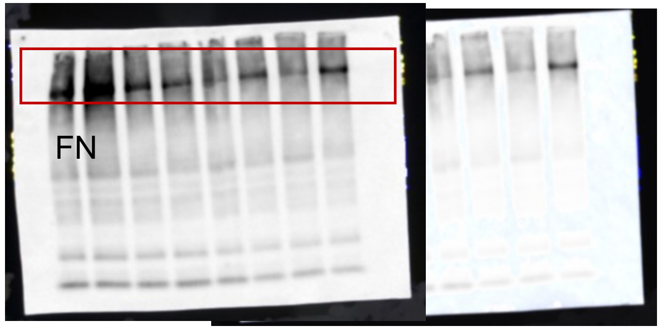

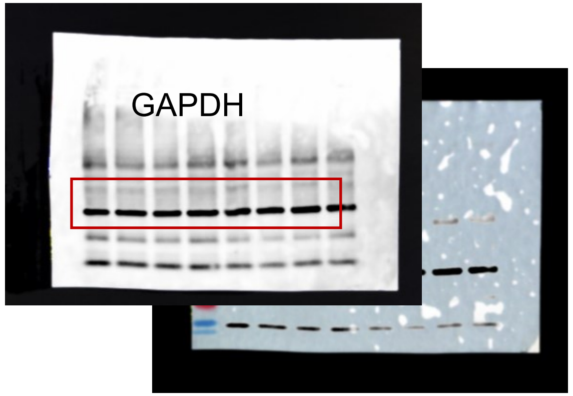

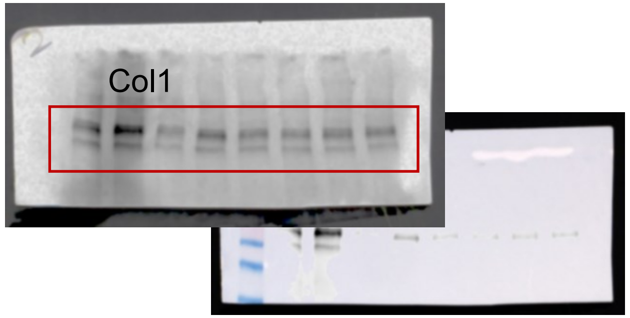

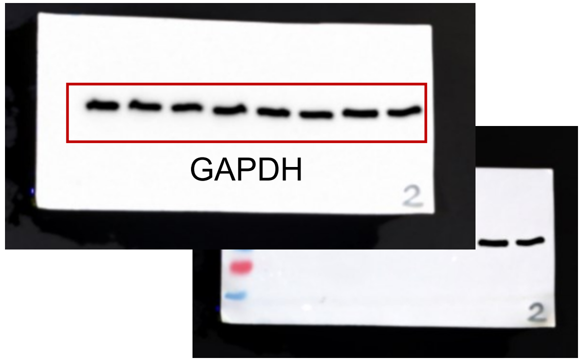

Figure 4B

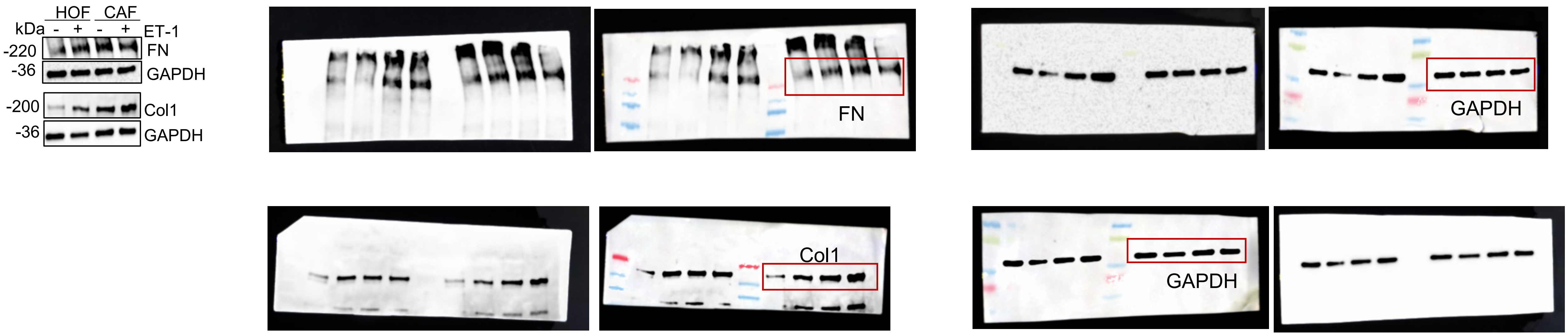

Figure 4C

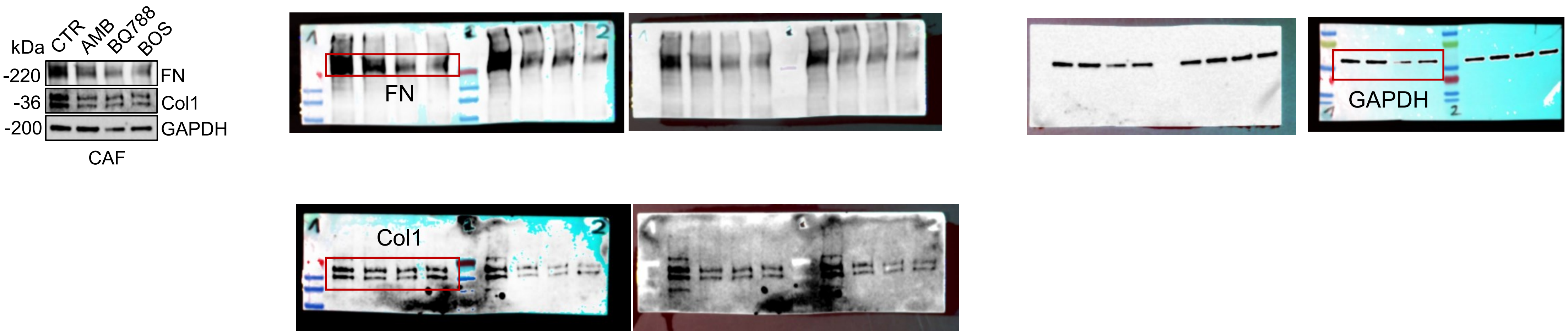

Figure 5B

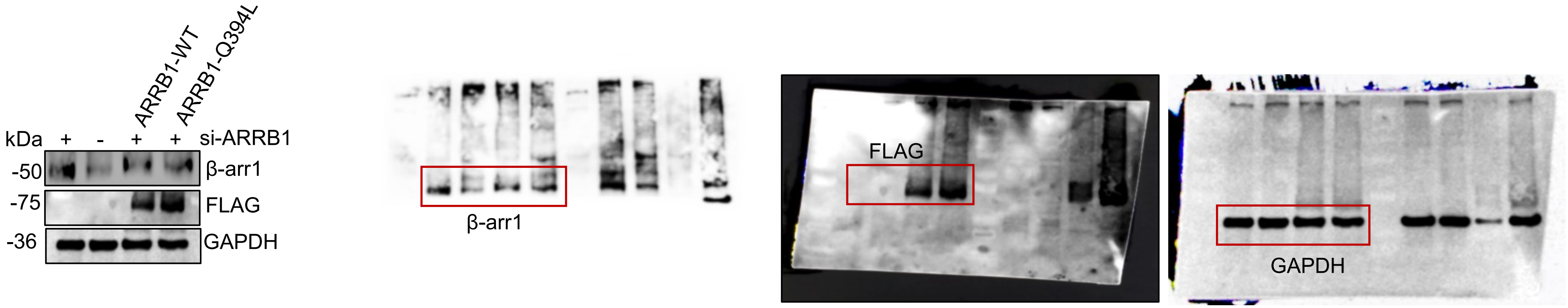

ARRB1-WT
ARRB1-Q394L
kDa
+
-
+
+
si-ARRB1
-50
β-arr1
-75
FLAG
-36
GAPDH
β-arr1
FLAG
GAPDH

Figure 7B

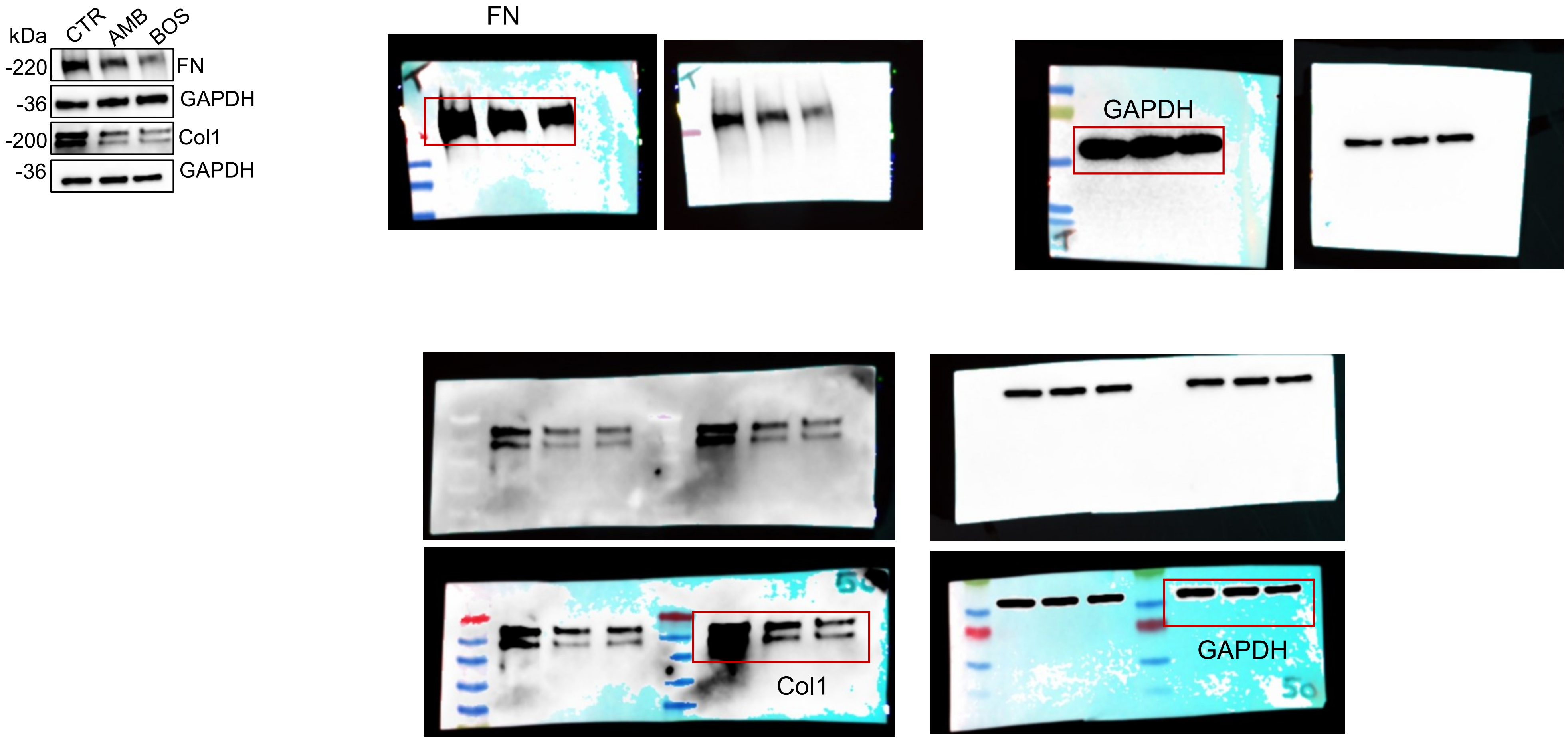
kDa
CTR
AMB
BOS
-220
FN
-36
GAPDH
-200
Col1
-36
GAPDH
FN
GAPDH
Col1
GAPDH
