# Supplementary figures and images for: The extracellular matrix protein type I collagen and fibronectin are regulated by β-arrestin-1/endothelin axis in human ovarian fibroblasts
Source: J Exp Clin Cancer Res. 2025 Feb 21;44:64. doi: 10.1186/s13046-025-03327-5 (PMC11844176; doi:10.1186/s13046-025-03327-5)

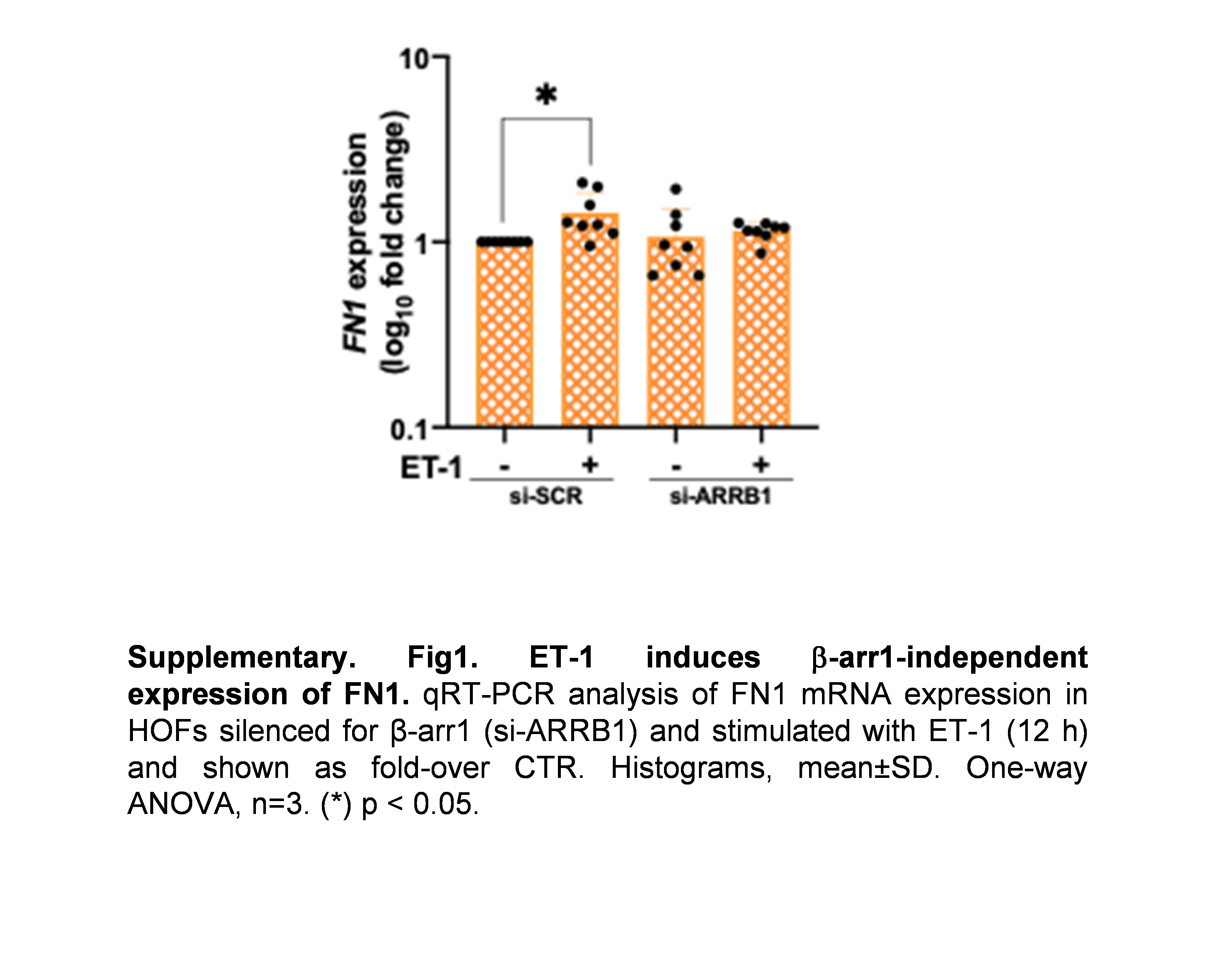

Supplement: Supplementary file 3 — Supplementary Material 3 [file 13046_2025_3327_MOESM3_ESM.tif]

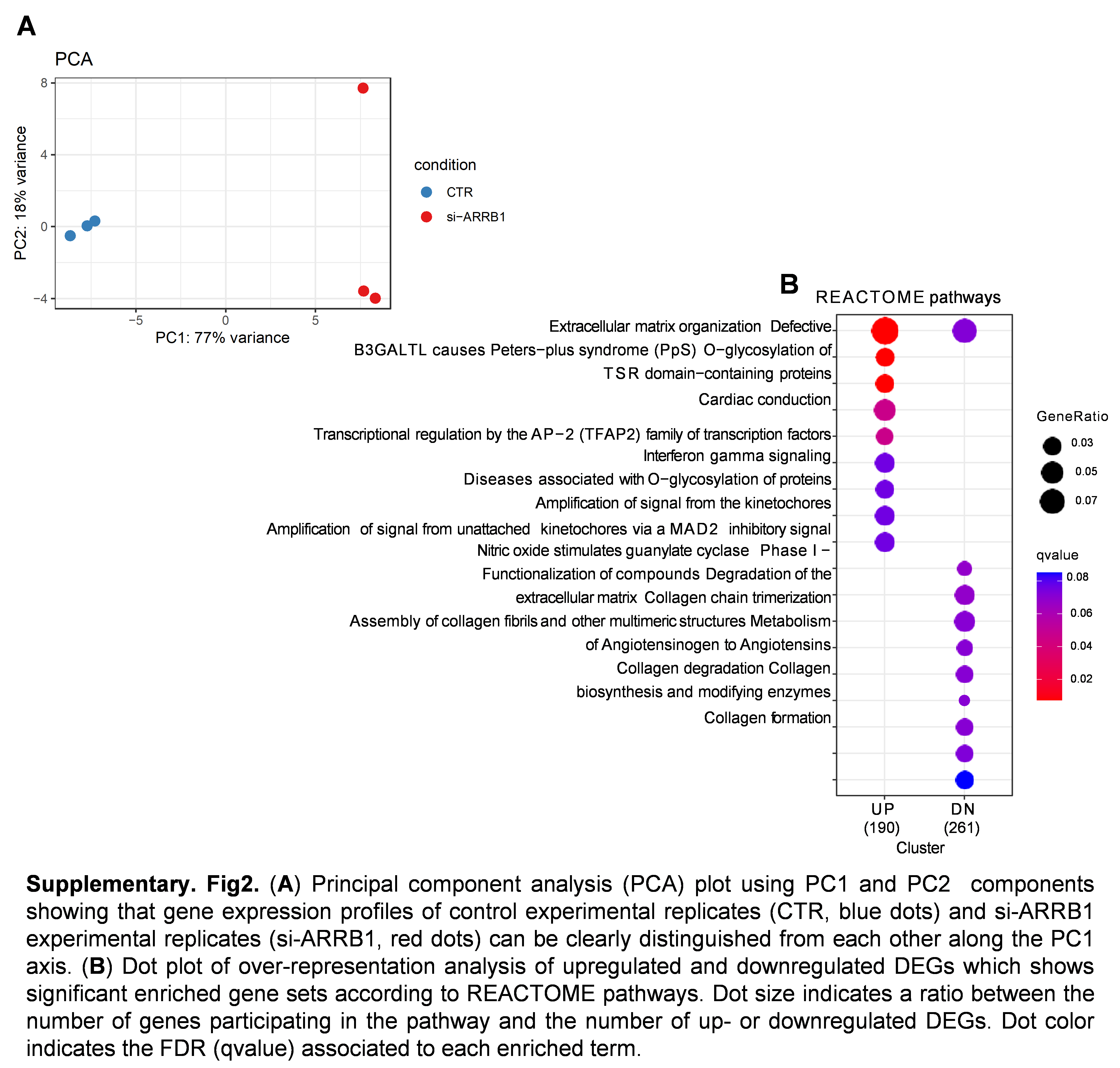

Supplement: Supplementary file 4 — Supplementary Material 4 [file 13046_2025_3327_MOESM4_ESM.tif]
